# Six-Month Pilot Testing of a Digital Health Tool to Support Effective Self-Care in People With Heart Failure: Mixed Methods Study

**DOI:** 10.2196/52442

**Published:** 2024-03-01

**Authors:** Alison Keogh, Carol Brennan, William Johnston, Jane Dickson, Stephen J Leslie, David Burke, Peter Megyesi, Brian Caulfield

**Affiliations:** 1 Insight Centre Data Analytics University College Dublin Dublin Ireland; 2 School of Medicine Trinity College Dublin Dublin Ireland; 3 School of Public Health, Physiotherapy and Sports Science University College Dublin Dublin Ireland; 4 Physiotherapy Department Beacon Hospital Dublin Ireland; 5 Cardiology Beacon Hospital Dublin Ireland; 6 Cardiac Unit Raigmore Inverness United Kingdom; 7 School of Medicine University College Dublin Dublin Ireland

**Keywords:** digital health, heart failure, cardiology, self-care, behavior change, eHealth, mHealth, mobile health, mobile app, mobile phone, elderly, self-care, self-management, digital tools, digital tool, human-centered design, app, apps, applications, wearables, wearable, Fitbit, usability, adherence, feasibility, congestive heart failure, cardiac failure, myocardial failure, heart decompensation

## Abstract

**Background:**

Digital tools may support people to self-manage their heart failure (HF). Having previously outlined the human-centered design development of a digital tool to support self-care of HF, the next step was to pilot the tool over a period of time to establish people’s acceptance of it in practice.

**Objective:**

This study aims to conduct an observational pilot study to examine the usability, adherence, and feasibility of a digital health tool for HF within the Irish health care system.

**Methods:**

A total of 19 participants with HF were provided with a digital tool comprising a mobile app and the Fitbit Charge 4 and Aria Air smart scales for a period of 6 months. Changes to their self-care were assessed before and after the study with the 9-item European HF Self-care Behavior Scale (EHFScBS) and the Minnesota Living with HF Questionnaire (MLwHFQ) using a Wilcoxon signed rank test. After the study, 3 usability questionnaires were implemented and descriptively analyzed: the System Usability Scale (SUS), Wearable Technology Motivation Scale (WTMS), and Comfort Rating Scale (CRS). Participants also undertook a semistructured interview regarding their experiences with the digital tool. Interviews were analyzed deductively using the Theoretical Domains Framework.

**Results:**

Participants wore their devices for an average of 86.2% of the days in the 6-month testing period ranging from 40.6% to 98%. Although improvements in the EHFScBS and MLwHFQ were seen, these changes were not significant (*P*=.10 and *P*=.70, respectively, where *P*>.03, after a Bonferroni correction). SUS results suggest that the usability of this system was not acceptable with a median score of 58.8 (IQR 55.0-60.0; range 45.0-67.5). Participants demonstrated a strong motivation to use the system according to the WTMS (median 6.0, IQR 5.0-7.0; range 1.0-7.0), whereas the Fitbit was considered very comfortable as demonstrated by the low CRS results (median 0.0, IQR 0.0-0.0; range 0.0-2.0). According to participant interviews, the digital tool supported self-management through increased knowledge, improved awareness, decision-making, and confidence in their own data, and improving their social support through a feeling of comfort in being watched.

**Conclusions:**

The digital health tool demonstrated high levels of adherence and acceptance among participants. Although the SUS results suggest low usability, this may be explained by participants uncertainty that they were using it fully, rather than it being unusable, especially given the experiences documented in their interviews. The digital tool targeted key self-management behaviors and feelings of social support. However, a number of changes to the tool, and the health service, are required before it can be implemented at scale. A full-scale feasibility trial conducted at a wider level is required to fully determine its potential effectiveness and wider implementation needs.

## Introduction

Heart failure (HF) is a major global cause of disability associated with high morbidity and mortality, frequent hospitalization, high health care costs, impaired functional status, and poor quality of life [1-3]. Defined as “a clinical syndrome with symptoms and/or signs caused by a structural and/or functional cardiac abnormality and corroborated by elevated natriuretic peptide levels and/or objective evidence of pulmonary or systemic congestion” [4], symptoms include shortness of breath (dyspnea), fatigue, pulmonary edema, and a reduced ability to complete activities of daily living [2]. Self-care behaviors are critical components of the long-term management of HF [3,5-8]. Self-care is an overarching concept formed of the key concepts of (1) self-care maintenance (eg, taking or adjusting medication as prescribed, engaging in physical activity, and adhering to a healthy diet), (2) self-care monitoring (eg, regular weighing), and (3) self-care management (eg, changing diuretic dose in response to symptoms) [5]. Adequate self-care requires patients to understand what they need to do, have the skills to implement advice given to them, and adjust their behaviors according to how they feel and their symptoms. This is a complex, multicomponent set of behaviors that is constantly evolving, which can result in patients finding it difficult to successfully undertake [2,3,9,10].

Consequently, recent years have seen growing interest in the development of new and novel ways to support patients in their self-care behaviors. In particular, digital health options have begun to be widely used as the ubiquitous use of smartphone apps, and wearable devices appear to be adopted to support health care [3,11]. Used independently, or in combination, such digital health tools (DHTs) may support patients to monitor key behaviors, support improved autonomy in their own care, and provide pathways for patients and health care professionals (HCPs) to communicate [2,9]. However, despite the promise of DHTs in the management of HF, results regarding its potential effectiveness are mixed [3,12,13]. The pace of development of digital tools has resulted in them only being tested in small numbers or over a short duration, and critically, these tools are rarely developed with clear clinical or patient perspectives embedded within them [3]. Recently, the use of human-centered design approaches has led to the successful development of digital health technologies designed to support chronic disease management [14]. As such, implementing a human-centered design approach to the development of DHTs designed to promote self-care behaviors in patients with HF may positively influence the impact their condition has on their quality of life.

Therefore, we used a human-centered design process, as described in the International Organization for Standardization 9241-210:2019 regulations [15], to design and develop a DHT to support effective self-care behaviors in people with HF over a medium-term duration of 6 months to test for the potential of participant fatigue with the DHT (ie, reduced usage over time, poor acceptability, etc). The full process and approach taken were previously outlined in detail elsewhere and pilot-tested over a 2 week period [16]. Specifically, a consumer grade device was used to understand whether such ubiquitous tools can be used to empower people with HF to self-monitor their condition, bridging the gap between them and their clinicians. However, this tool required a more robust assessment of the longitudinal impact on self-care behaviors. Additionally, we wished to get an initial indication of its potential use from the perspective of the HCPs. Therefore, the aim of this study was to conduct a 6-month observational pilot test of this DHT in practice to examine its usability and the participant’s adherence to the system.

## Methods

### Recruitment

Participants were recruited from a private hospital in Dublin, Ireland, between July and October 2021 and had previously been diagnosed with HF. Purposive sampling was used using the patient lists of Beacon Hospital Cardiology to facilitate the assessment of the acceptability and usability of the DHT. Participants were deemed eligible if they could provide written informed consent; were previously diagnosed with HF; were under the care of Beacon Hospital Cardiology (aged ≥18 years); were under New York Heart Association classification 1-3; were open to the use of technology in the promotion of HF self-care; had access to an internet connection or mobile data; and were intellectually, visually, and auditorily capable of communicating with the investigator and understanding and complying with the requirements of the study. Participants were deemed ineligible if they were medically unstable or undergoing medical treatment judged not to be medically compatible by the investigator (eg, undergoing treatment for cancer), or if they had any skin condition that may affect the integrity of their skin when wearing the activity tracker. Participants (n=43) were approached directly by the members of the cardiology team to determine their interest and eligibility in the study.

### Ethical Considerations

The study received ethical approval from the Beacon Hospital Research Ethics Committee (BEA0114 and BEA0151), and written informed consent was obtained from all participants before commencing the study. No financial compensation was provided to participants taking part.

### Digital Health Tool

**Figure 1:**
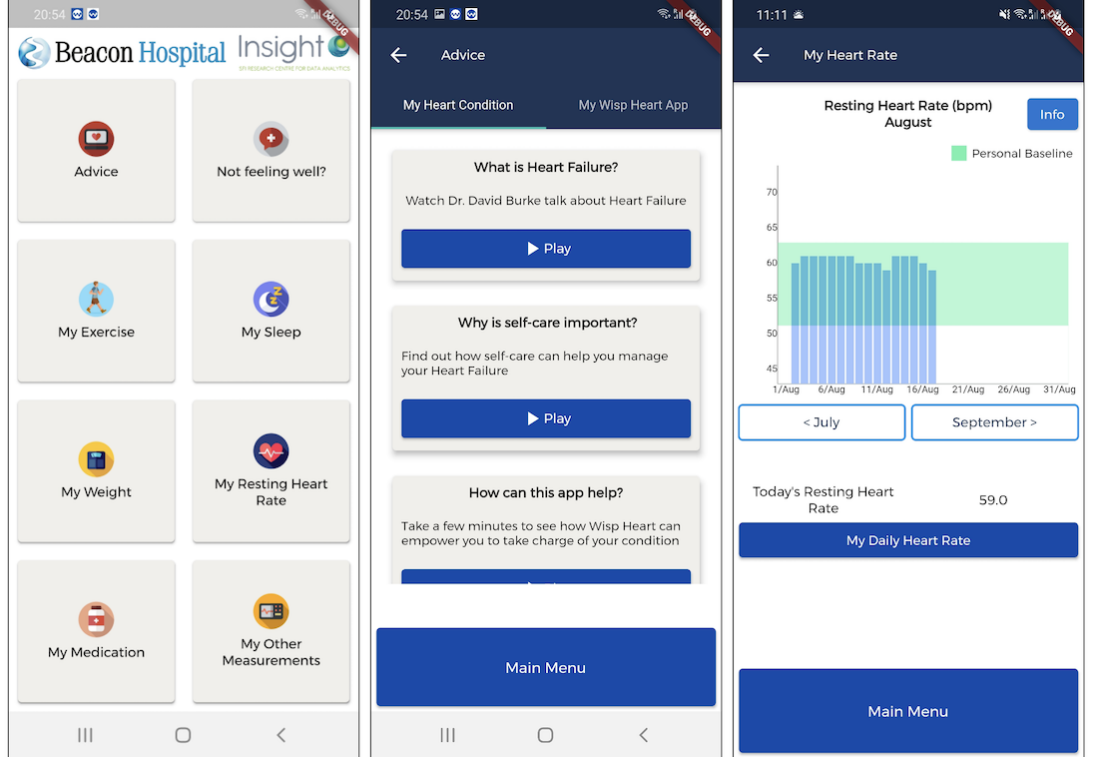
Screenshots from the mobile app detailing the main menu, advice section, symptom report, and screens [16].

The DHT used in this study was designed using human-centered design steps and included behavior change techniques, as previously outlined in detail by Johnston et al [16]. Briefly, the system was designed to comprise a cross-platform (iOS or Android) mobile app capable of linking to a consumer activity tracker and smart scales, specifically, the Fitbit Charge 4 and Aria Air smart scales (information also available in [17]). The mobile app was broadly divided into five sections: (1) advice, (2) symptom reporting, (3) activity tracker and scale data (exercise, weight, heart rate, and sleep), (4) medication reminders, and (5) other vital sign tracking—all targeted through the inclusion of specific behavior change techniques (Figure 1) [16,17]. During the design of this DHT, an initial prototype was trialed with participants with HF for a period of 2 weeks, where positive feedback and adherence were seen [16]. The system was considered easy to use, positively affected their motivation to engage in key self-care behaviors, provided them with skills and perceived knowledge that made them more aware of the importance of self-care behaviors, positively influenced their confidence, and facilitated help seeking. After this, aspects of the system that needed to be improved were identified. These changes were implemented before deployment in this longer study. Specifically, the scaling in the app was adjusted to support larger fonts, the ability to input decimal points for vital signs was inputted, screens were not allowed to take a time-out while videos were playing, the information button was made more visible, technical issues surrounding daily data were addressed, and the ability to visualize within day heart rate data was implemented [16].

### Study Methods

**Figure 2:**
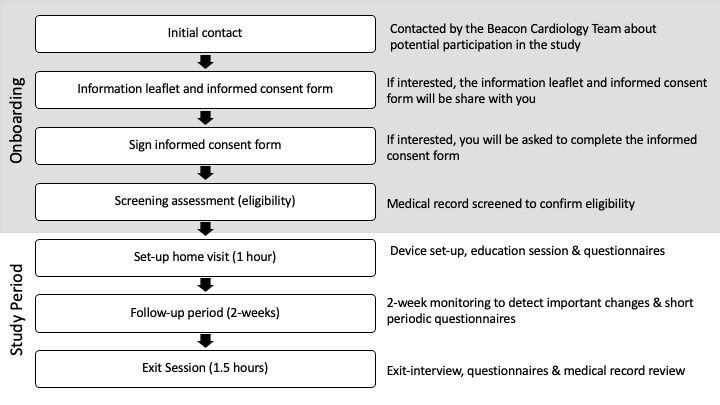
Study flowchart.

The recruited participants were invited to an initial setup session at the hospital (Figure 2). Demographic data such as age, sex, and the highest level of education were collected at the beginning of the session. The participants then completed the 9-item European HF Self-care Behavior Scale (EHFScBS) [18,19] and the Minnesota Living with HF Questionnaire (MLwHFQ) [20,21] to evaluate self-care behaviors in patients with HF and the effect of HF treatments on the quality of life.

Following a setup and familiarization session with WJ (approximately 40 minutes), participants were asked to use the system as part of their usual daily routine for the following 6 months. Depending on their recruitment date, participants were using the DHT for a 6-month period between July 2021 and April 2022. During this period, patients were asked to wear the Fitbit Charge 4 activity tracker on their wrist, take their weight every morning using the Fitbit Aria Air scales, and interact with the developed mobile app. A *check-in* symptom questionnaire was completed once a month [16]. This same questionnaire was also triggered to be sent to participants once any of their monitored components (ie, heart rate, sleep, weight, or physical activity) changed by 2 SDs from their baseline level in the previous 7 days. In the event that a trigger occurred, the questionnaire was sent to participants as an alert in the app, informing them of a change and asking them to complete the questionnaire. The questionnaire was then sent, along with a trigger, to the cardiology team of the Beacon Hospital who would telephone the participant to determine whether any further medical action or intervention was required.

At the end of the 6-month period, individual semistructured interviews were completed over the phone and recorded with each participant. Open-ended questions were used to explore their perceptions of the acceptability, usability, and practicality of the DHT; understand their experiences pertaining to the impact of the DHT on their self-care behaviors; identify usability and user experience issues; and identify aspects that could improve the DHT (Multimedia Appendix 1). Participants also completed 3 usability questionnaires: System Usability Scale (SUS), a questionnaire designed to measure system usability [22]; Wearable Technology Motivation Scale (WTMS), a questionnaire based on the intrinsic needs listed within self-determination theory [23]; and the Comfort Rating Scale (CRS), a questionnaire designed to assess the comfort of wearable devices across the dimensions of emotion, attachment, harm, perceived change, movement, and anxiety [24]. They also repeated the EHFScBS and MLwHFQ to indicate whether a change in their behaviors or quality of life occurred.

After the completion of the study, semistructured interviews were also conducted with 2 members of the clinical team in Beacon Hospital Cardiology to explore their perceptions of the DHT (Multimedia Appendix 2).

### Data Analysis

The recorded interviews were transcribed verbatim and anonymized. The coding of participants’ experiences with the DHT over the 6-month period was done using the Theoretical Domains Framework (TDF). The TDF was used within the development study to map the target behaviors of the tool; thus, it was used as a lens through which to view the usability, adherence, and potential behavior changes noted during this 6-month study. A deductive content analysis was undertaken whereby transcripts were coded according to the components of TDF [25,26] using a critical realist approach. This approach posits that a reality exists independent of our construction of it, while maintaining that our knowledge of it is interpretive, partial, and fallible [27]. In taking this approach, we recognize that our own experiences influence our insights but that we maintain our objective to view the situation as it occurs. To elaborate on our experiences, AK is a research physiotherapist with over 5 years of experience in digital health research and a PhD in behavior change. CB is undertaking a PhD in behavior change, with expertise in the use of the TDF and publications using the same. Together, these experiences influence and enrich their interpretation of the data.

AK coded all transcripts. First, they familiarized themselves with the data by reading and rereading all transcripts and generating initial notes on the data. Meaningful phrases were highlighted and assigned codes according to the domains of the TDF [28,29]. CB then acted as a critical friend to the coding, reviewing 20% of transcripts and providing critical feedback to improve the interpretation of findings and discussion of codes. How these barriers and facilitators align to each of the key skills of self-management was considered to identify areas for further development. Finally, given that only 2 HCPs were interviewed regarding their experiences, information from their transcripts was summarized narratively.

Questionnaire data were analyzed using SPPS Statistics for Mac (version 27; IBM Corp). The questionnaire data were scored using the appropriate standardized procedure for each questionnaire. The MLwHFQ is scored by summing each of the components, resulting in a score ranging from 0 to 105 (whereby higher scores indicate higher impairment) [20]. The EHFScBS 9-item is scored by reversing the responses to the questionnaire and standardizing them [30]. This results in a score ranging from 0 to 100 (where a higher score indicates good self-care, and <30 is deemed as inadequate) [18]. Changes in results before and after the study were measured using a Wilcoxon signed rank test. The *P* value was set at .05; however, this was adjusted with a Bonferroni adjustment whereby *P*<.03 was significant. The SUS is scored out of 40 but converted to a 0-100 scale as per the standard procedure, with >68 deemed acceptable and >80 considered excellent [31]. Each item of the CRS is scored from 0 to 20 (where higher scores equate to worse comfort) [24]. The median of the 6-item questionnaire was calculated. Finally, the WTMS is scored by calculating the average score across the different components for each participant, resulting in a score ranging from 0 to 7 (whereby 7 indicates higher intrinsic motivation) [23]. In addition, adherence was determined by identifying the number of days a user wore the Fitbit device throughout the day and recorded their weight.

## Results

### Participants

A total of 43 people were contacted by the clinicians in the Beacon Hospital to invite them to participate. Of these, 10 (23%) were not contactable, 1 (2%) did not satisfy the inclusion or exclusion criteria, 6 (14%) declined, and 7 (16%) did not use a smartphone. A total of 19 (44%) participants were recruited, of whom 17 completed the poststudy follow-up session (Table 1). One participant withdrew in the middle of the study because of increased health concerns, and 1 participant was unable to be contacted at the end of the study. Results regarding participants acceptability and changes to their self-care routines are based on the 17 who completed the entire study. Participants wore their devices for an average of 86.2% of the days in the testing period ranging from 40.6% to 98% (average of 157 days out of a possible 184 days). Furthermore, participants weighed themselves for an average of 73.7% of the potential testing days, ranging from 4.9% to 100% (average of 134 days out of a possible 184 days).

**Table 1:** Participant demographics (N=17).

Demographic		Value
**Sex, n (%)**		
	Male	11 (65)
	Female	6 (35)
Age (years), mean (SD; minimum-maximum)		72 (9.9; 54-81)
BMI, mean (SD; minimum-maximum)		28.3 (6.1; 19.3-40.4)
**Education level, n (%)**		
	Did not complete second level	2 (12)
	Completed second level	4 (24)
	Third level education (any)	7 (41)
	Not reported	4 (24)

### Patient-Reported Outcomes

**Figure 3:**
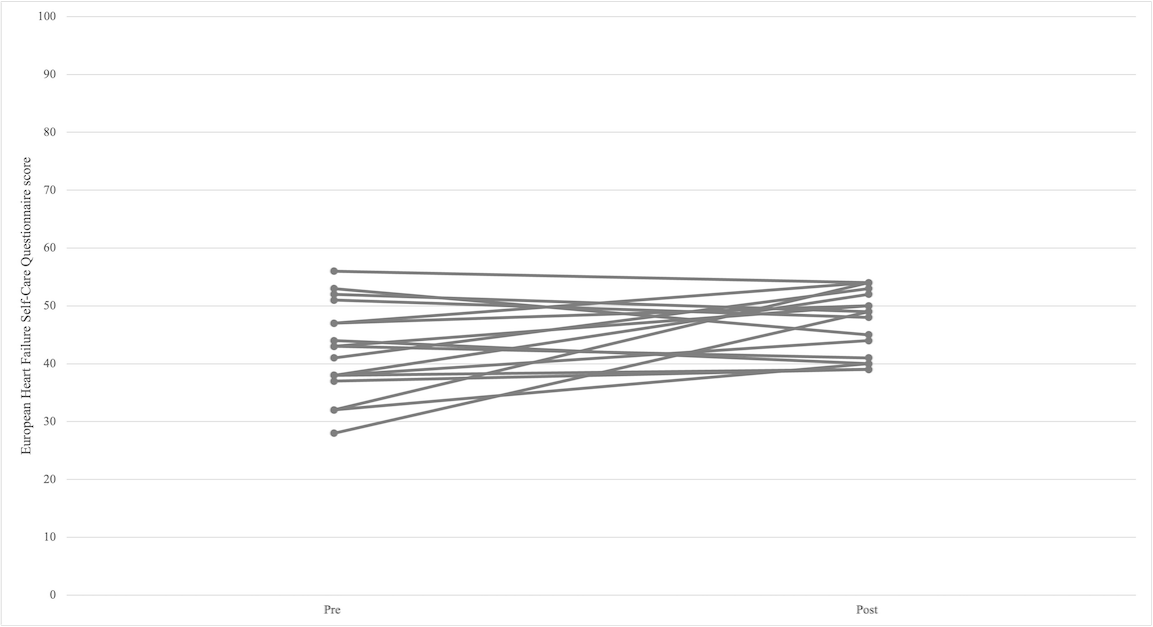
Pre- and post-results per participant for the European Heart Failure Self-care Behavior Scale.

**Figure 4:**
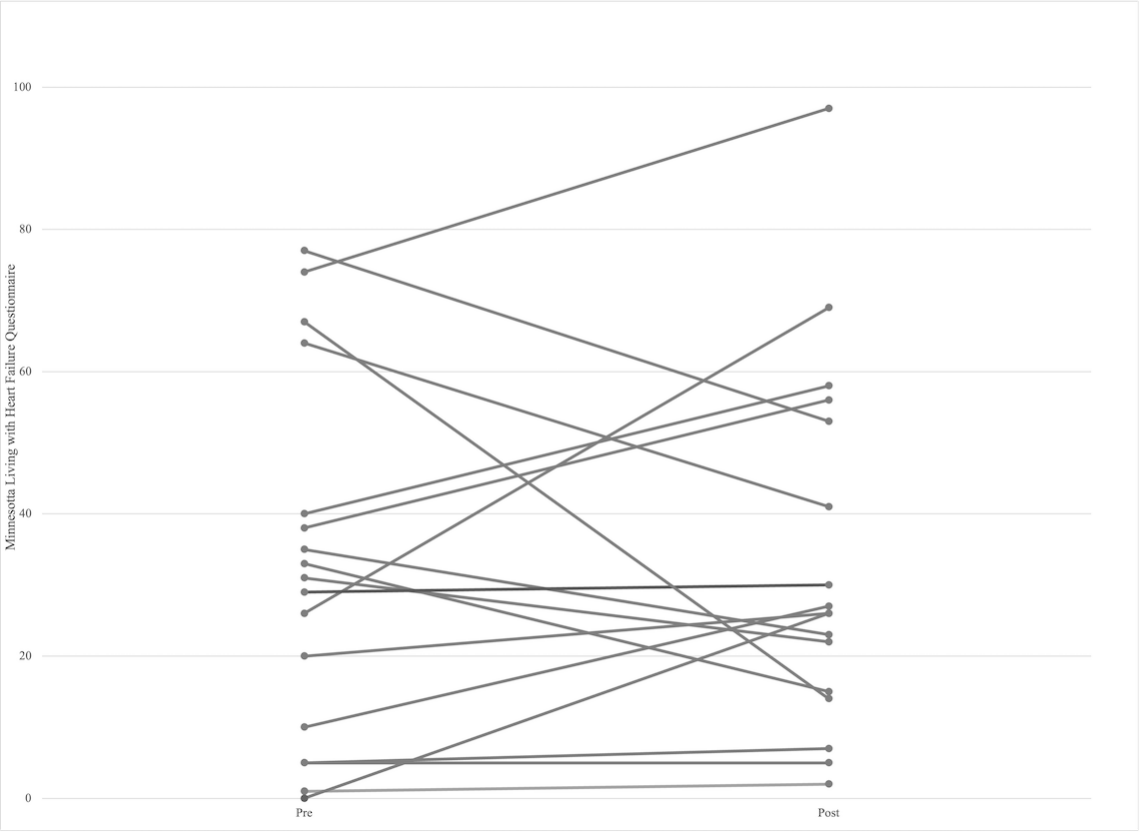
Pre- and post-results per participant for the Minnesota Living with Heart Failure Questionnaire.

Although improvements in the EHFScBS were seen between baseline and the completion of the study, these changes were not significant (*P*>.03; Table 2, Figures 3 and 4). No participants were deemed to have inadequate self-care according to this scale. Similarly, although improvements were seen in the MLwHFQ results, these were not significant. Nonetheless, the median score suggests moderate quality of life, with 7 participants demonstrating good quality scores (41%) and 5 listing poor scores (29%) [32].

With regard to the acceptability of the DHT, Table 3 lists the results from the SUS, WTMS, and CRS. The SUS score considered the Fitbit and mobile app as a whole system. Results suggest that the usability of this system was not acceptable to participants as the median score was 58.8 (55.0-60.0; 45.0-67.5). Indeed, no participant scored the system above 68, which is considered to be the threshold of acceptability. In contrast, participants demonstrated a strong motivation to use the system according to the WTMS (median 6.0, IQR 5.0-7.0; range 1.0-7.0), whereas the Fitbit was considered very comfortable as demonstrated by the low CRS results (median 0.0, IQR 0.0-0.0; range 0.0-2.0).

**Table 2:** Changes in participants’ reported self-care of their heart failure (HF).

Questionnaire	Median prestudy results (IQR; minimum-maximum)	Median poststudy results (IQR; minimum-maximum)	*Z* score	*P* value
9-item European HF Self-care Behavior Scale (0-100)	52.8 (38.0-47.0; 22-89)	72.2 (41.0-52.0; 42-94)	–1.61	.11
Minnesota Living with HF Questionnaire (0-100)	31.0 (10.0-40.0; 0-77)	26.0 (15.0-53.0; 2-97)	–0.39	.70

**Table 3:** Participant reported acceptability of the digital health tool.

Questionnaire	Median results (IQR; minimum-maximum)
System Usability Questionnaire (0-100)	58.8 (55-60; 45.0-67.5)
Wearable Technology Motivation Scale (0-7)	6.0 (5-7; 1.0-7.0)
Comfort Rating Scale (0-20)	0.0 (0.0-0.0; 0.0-2.0)

### Participant Interviews

**Figure 5:**
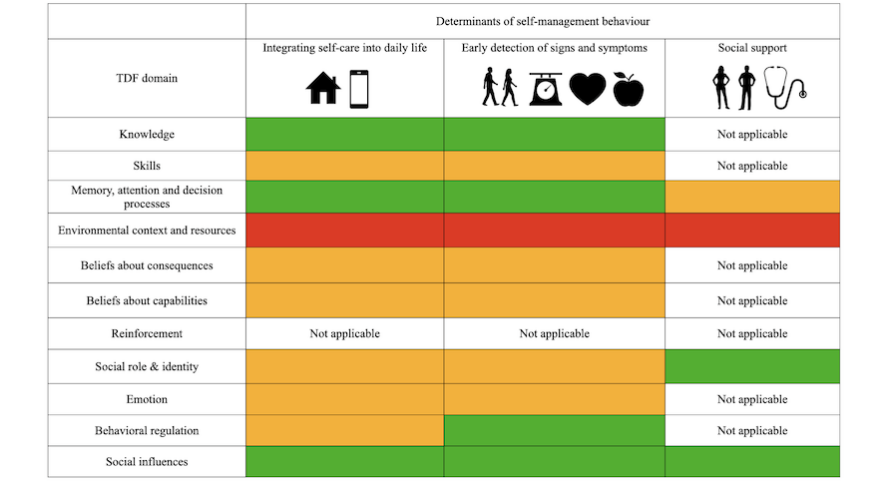
Demonstrated impact of each Theoretical Domains Framework component on self-management of heart failure (green = facilitator, orange = unclear, red= barrier).

A total of 13 of the TDF domains were viewed as barriers or facilitators to the self-management of HF, whereas just 1 domain of the TDF did not match data (“optimism”). However, alongside “goals” this domain was not originally mapped to the key behaviors of self-management during the development of the mobile app [16]. A limited number of codes were noted for “goals” and “reinforcement,” suggesting that neither domain plays an important role in the facilitation of self-management in HF; therefore, they were excluded from the analysis. Of the remaining domains, 5 were generally viewed as facilitators of self-management (knowledge; social role and identity; memory, attention, and decision processes; social influences; and behavioral regulation). One (environmental context) was a barrier to self-management, whereas 5 were neither (skills, beliefs about capabilities, beliefs about consequences, intentions, and emotions; Figure 5).

#### Knowledge

Participants noted that the information provided in the mobile app was simple to understand and provided them with a greater general awareness of important elements to them and the management of their heart condition, specifically heart rate and weight. Indeed, people noted that, if anything, the information was too simple and they would have liked more personalized information, information about dietary requirements and blood pressure. Thus, overall, the knowledge provided by the app was considered useful in the management and awareness of their HF.

Well it was very simple to work. It kept me up to date. I kept an eye on my weight every morning which I usedn’t do. And then I would check the heart rate as well and then of course what I found very helpful was the questionnaire that you sent on every month.

P10, male, 80

From my own personal point of view, blood pressure, since I had open heart surgery, my blood pressure has been very low, so I suffer on a daily basis with light-headedness and all of that. So, I suppose I would have found it useful to know blood pressure readings.

P17, female, 67

Well, like as I said, it wouldn’t give me what the problem was with me, like the blood flow through the heart, it wasn’t doing that. It’s just monitoring the heart.

P2, male, 74

#### Memory, Attention, and Decision Processes

This domain is a complex element that included participants’ memory of completing self-management behaviors and their awareness and understanding of the data provided to them. For some, they were unclear as to what the green area within the graphs of the mobile app represented; thus, they paid little attention to it. Furthermore, participants remarked that they sometimes forgot to look at the app and thus were passively monitoring themselves. However, participants were positive about the fact that the app provided them with a way to become more aware of their daily patterns. This, they believed, supported their self-management as it helped them to know they were remaining consistent. Specifically, they were comfortable if they went outside of their normal range for 1 or 2 days but knew to contact someone if that became persistent. This suggests that people did not explicitly change their behavior, but simply being aware that everything was “normal” was sufficient for them to feel confident in themselves.

I knew that I would be contacted if there were discrepancies or going outside the zone, so I knew that, but I mean if for example my weight had started to increase, I would have been very aware of checking up on fluid retention and all that sort of thing, yeah. It was good to be able to see that, I wouldn’t, honestly, have been aware, unless my ankles, I’ve never had particular puffiness or anything like that or similar, so, but, I think there was a slight bit of that whenever I was in hospital in October, although it wasn’t obvious, it wasn’t visible but obviously it would have been obvious from a daily weight reading.

P17, female, 67

#### Behavioral Regulation

The use of the digital tool facilitated participants to weigh themselves (daily, weekly, or biweekly). This was the most active behavior change that appeared to occur as a result of the system. Some reported being motivated to go for a short walk if they felt that they had not reached enough steps according to what their DHT was; however, overall, people reported not changing anything substantially. As previously mentioned, an awareness of their normal appeared to be the greatest benefit to them. This may be because other features such as monitoring their sleep and heart rate were mostly passive in nature. The components that required the most active engagements (ie, medication adherence and other methods of monitoring) were not used by any participant as they felt that they already had a method to track their medication that worked for them. Essentially, the digital tool appeared to integrate easily into their existing methods of regulating their HF, supporting their ability to monitor their key metrics without requiring too much additional effort on their part.

I’ll certainly keep the Fitbit up…wearing the Fitbit, I will keep that going definitely, so that I can keep an eye on things. I won’t say you can become obsessed with it but it gives you a good handle, I wouldn’t be checking my heart rate every couple of hours or anything like that but every couple of days I would do or if I did something strenuous I would just have a look and see how am I and what I am doing or anything else like that.

P3, male, 68

### Social Influences

Participants continue to rely on HCPs to lead the management of their condition and initiate topics of discussion. Furthermore, they were motivated to join the study as a result of their doctor asking if they were interested in it. Their perception was that if the doctor felt that it might be useful or interesting, then they were happy to participate. Despite this deference to HCPs, some felt that the information from the DHTs would empower them to talk to their doctors about their progress. However, the strongest element of support that they received from the tool was the comfort they gained in knowing they were “being watched.” Participants appreciated the calls that they received from HCPs if the system was triggered. Far from feeling intruded on, they instead felt supported and “minded” from afar, and had no privacy concerns regarding these triggers.

I have to say, I really appreciated it very, very much. I can’t really think of a particular negative. It really helped me and really reassured me because I had two episodes last year where I went into atrial fibrillation and you know I think it was good to know that, you know, if I did go off the baseline and someone contacted me, that they would then offer that I could speak to someone on the cardiac team, because sometimes it’s very difficult to access even your cardiologist, you know? Even getting past the secretary can be very difficult, so that was, yeah, I really liked that. I just felt that, you know, if something did go drastically wrong that someone was there picking up on it.

P17, female, 67

I think it’s nice the idea that if something goes badly wrong that somebody rings up like a few times my heartbeat has changed and I’ve got phone calls to ask am I ok and trying to figure out why.

P4, male, 64

#### Social Role and Identity

Participants perception of themselves and their own identity appeared to facilitate their self-management behaviors. This was closely linked to knowledge and memory, attention, and decision processes. Specifically, being aware of their data and their patterns of behavior sparked participants to reflect on “how lazy I am one day and how much I am doing the next day type of thing.” This suggests that they saw it as important to understand what they can do to help themselves, which was facilitated by the DHT. Furthermore, participants described being diligent about going to the doctor regularly and “doing everything they were saying I should do,” in order to help themselves manage their condition. Thus, they recognized the role that they had to play in their own condition, even if that is being led by the cardiologist.

#### Skills

Although skills were related to participants’ ability to self-manage, in this study, they were also required to interact with the digital tool in order to do so. Indeed, the biggest barrier to them being able to self-manage with technology was the technology itself. Specifically, for those less familiar with technology, it took them a while to settle into its use. Some faced issues with syncing and connectivity, for which they required support from family members or members of the study team to overcome. However, as they got used to it, and as problems were solved, they then found that the DHT was an enabler to their self-management as it became one place for them to see all of their information.

Technology, you know it was always a bit beyond me a little bit. So, I thought it worked out fine ….I became a little more comfortable with it. There were a few times alright….and again with the weighing scales, there was a problem and I made contact with one of your colleagues. And simple things like for example on the tablet, I tend to just ….just press the on/off button until the screen goes out you see.

P16, male, 81

#### Beliefs About Consequences

The key for this was whether participants believed that there was a link between their self-management behaviors and their HF. For example, 1 participant did not see the relevance of weighing themselves as they could not see how it impacted their HF. In contrast, others were using the app to help support their perception for how they were feeling. In general, people were able to look at their data and explain any discrepancies as a result of their recent behavior; thus, the system appeared to support building this belief. However, it was not always clear how this linked explicitly to their heart condition, as opposed to their overall health.

Well I’ll tell you once or twice it went down [their weight] and I said ‘Oh my God am I not well’ and then the next day is would come back up. Now I am only talking about a pound here or a pound there but if it did go up oh I would, I’d certainly have to watch, cut out maybe eating a bar of chocolate which I eat every so often or a sweet or whatever. I would be aware of it.

P8, female, 81

#### Beliefs About Capabilities

Some participants felt they did not possess the skills to get the most out of the DHT. However, their uncertainty was also related to their ability to self-manage themselves in general. One person was wary about traveling in case something went wrong; another simply listed being unmotivated to self-manage. In contrast, others felt that the DHT improved their capabilities by providing them with the information to make informed choices and to speak to their doctor if needed.

I probably am not tech-savvy enough to have gotten everything I needed to get from both of those or everything they could give me, and I’m also going to, in the early part of the interview, put my hands up and say that I didn’t probably put enough effort into that.

P18, male, 68

I feel good at the minute, I feel ok, and everything’s going well, and I’m going swimming, I’m going walking, I don’t feel any different up in the heart…...I wouldn’t have known all this stuff if I hadn’t of had the monitor on me and the whole lot, so I think with all this it’s good.

P14, male, 64

#### Emotion

The biggest change in emotions reported by participants was the confidence they had in managing themselves as a result of the DHT, and the reassurance they received knowing that they were being monitored. A feeling of safety was reported as a result of this. Despite this, some participants reported moments of anxiety or alarm if the DHT sent them a trigger unexpectedly, or if their data were outside of their normal. For one person, it simply took them some time to realize that a certain amount of change is considered normal; thus, it ultimately led to them feeling reassured once they learnt this. Others though would feel guilty if their activity levels were low, or if they were not losing weight.

Well, I am not as fretful now as it was at the beginning and I do think that wearing the Fitbit has been part of that – knowing that there is something there and also the fact that I was contacted on several occasions that my baselines had changed in a few things.

P7, male 54

It made me anxious because I looked back on a night like that and say well, I can’t see anything that that’s gone terribly astray here. I would like to have known what triggered it.

P9, female, 71

#### Environmental Context and Resources

The focus of this domain was on factors within the environment that either supported or hindered participants’ self-management. In relation to the DHT, having to charge the Fitbit was a downside, but not a burden. Issues connecting the scales were a greater burden as it may result in incorrect readings and were difficult for participants to fix. Other elements that acted as barriers to self-management were typically related to life events, for example, other illness, bereavement, the weather, relaxing their diet while on holidays, and the area where they live. In addition, participants’ medication was noted as negatively impacting their sleep or weight. It was unclear whether this was medication specific to their HF or whether it was related to other comorbidities, but regardless, it influenced elements of their self-management behaviors that they were tracking.

I suppose it was a bad time of the year in the sense, by the time I’d get home from work in the evening, it was pitch dark. So, I wasn’t getting out for a walk and that. We only get kind of a half an hour lunch break. So, you didn’t even have time [to exercise].

P15, female, 55

Well, weather wise and then, you know, we had an awful lot to do after the funeral, you know... things had to be sorted, well they are not even sorted yet but anyway, you know, things had to be done.

P1, female, 81

### HCP Experiences

The 2 HCPs (1 consultant cardiologist and 1 physiotherapist) were broadly positive around the potential for this DHT to support HF management; however, they felt that this would require a number of changes both to the system itself and to how it is implemented before its utility is realized.

Both HCPs were surprised at the level of “hands-on” work required to manage the DHT and the triggers sent through it. Essentially, they expected that the trigger system would reduce the work of clinicians, whereas, in reality, it simply transferred some of the work to behind the scenes data management, or even increased their work. Specifically, the system of triggers required them to spend a number of hours each week calling participants and following up on those who did not answer. However, as the purpose of this study was to explore the participant perspective, the HCP did not monitor the number of triggers received or the number that resulted in a hospital visit. Nonetheless, it was felt that future iterations of this tool need to set different criteria for when a participant was called or not.

They felt that it was only feasible to recruit participants with greater health and digital literacy, and those who were based locally to support them in the initial setup of the DHT. However, neither of these elements were explicitly listed as inclusion or exclusion criteria within this study. Recruitment was undertaken using a purposive, pragmatic approach whereby participants were recruited from the existing patient list of cardiologists within the Beacon Hospital. The HCPs acknowledged that their perceptions of a person’s awareness of their condition and their ability to use technology were born out of their interactions with these patients and not from objectively measured assessments. In general, they did not approach patients who they considered to be more frail and older, who appeared to have greater anxieties around technology, and who were less aware of their condition.

Ultimately, it was felt that the DHT has great potential, but that it would require significant changes within the health care service if it were to be implemented into practice. Specifically, adjusting the trigger thresholds, altering the protocol for calling people, and ensuring that the HCPs have dedicated time in their schedules to manage these digital data are critical for its progress.

## Discussion

### Principal Findings

This 6-month observational study assessed the feasibility and utility of our human-centered designed DHT to evaluate user engagement and observationally evaluate whether it may affect end points such as quality of life and self-care behaviors. Similar to the results of our development paper [16], participants demonstrated high adherence to the DHT and reported increased awareness of their behaviors and increased confidence in themselves. Questionnaires demonstrated that it was a comfortable device and that their motivation to wear the Fitbit was high, although the usability of the DHT was considered poor. However, interviews suggest that this result may be linked to their low confidence regarding the use of technology. Participants were most impressed at the trigger function of the DHT, which provided them with a great sense of safety and comfort.

### Comparison With Previous Research

Self-care refers to “performing the daily activities that serve to maintain or restore health and well-being, prevent illness, and manage chronic illness” [33] and includes the knowledge, skills, self-efficacy, and attitudes required to effectively manage signs and symptoms as they arise [10]. Similar to previous research, participants in this study reported that they take their medication as prescribed, have lower levels of physical activity than desired, and monitor their weight regularly but not daily [6]. Notably, their awareness of their sleep, weight, activity, and heart rate increased; however, they felt that they did not change anything substantially as the result of this information, while their EHFScBS and MLwHFQ results remain unchanged. This may be because the included participants were not considered inadequate in their self-management at the start of the study; thus, it should be questioned whether they needed to change their behavior unless they began feeling unwell. Furthermore, although changes were not significant, this may be the result of the small sample size, and nonetheless, positive changes in both outcome measures were noted. However, it should also be considered whether no changes occurred because participants were not clear as to why they were assessing certain elements. For example, many seemed to feel that monitoring weight was necessary because they needed to lose it, not because they were monitoring water retention, a finding similar to previous research [34]. Furthermore, skill building requires more than information alone and instead should focus on deficits and managing unique situations as they arise [34]. It should be considered whether, by relying on a trigger system, we are removing this skill rather than building it. However, given the complexity of such decisions [34,35], and the relief provided to participants as a result of the triggers, further thought should be given as to how skills can be developed alongside the support provided by triggers.

When we mapped the participant experiences to the elements of the TDF, the domains of knowledge, social influences, and social identity appear to be particularly strong in facilitating self-management. Although the study was meant to improve participants’ own abilities and motivation to integrate behaviors into daily living, according to the results of “social influences,” it is possible that they took part in the study and remained so adherent because of their desire to follow doctors’ wishes. This brings up a persistent issue with self-management and the motivation to complete it. Theories such as self-determination theory posit that people will initiate and maintain a behavior if it supports intrinsic motivation [36]. Participants began to note that they were able to keep an eye on themselves and that it was up to them to manage what they could control, suggesting that there is certain amount of intrinsic motivation generated as a result of being able to monitor themselves, and this is further supported by the results of the WTMS. However, relying on doctors’ advice to participate and adhere to an intervention is externally motivated behavior that risks poor engagement over time [36]. There is a fine balance between following advice in a partnership and following instructions [37,38]. Ultimately, the most effective and valued element of the intervention was the link that participants had with their HCP. Future studies should consider how to train participants to lead conversations in this area with their clinicians, so that they are more empowered to conduct effective self-care, rather than simply rely on triggers being sent to them. Furthermore, the emotive elements of self-management appear to have played a strong role in participants’ experience of the study. Specifically, the benefits were confidence and reassurance, whereas guilt and anxiety also prevailed. Previous research has suggested that emotional reactions such as fear or anxiety, which tend to be viewed as maladaptive coping strategies, may also have a positive influence on self-care [39]. Many patients describe action-based strategies such as learning how to “pace” their activities or “listen to their bodies” to help optimize their ability to maintain physical activity [39]. Seeking guidance and advice from trusted sources is an integral component of self-care, and knowing when to ask for help is considered to be a tactical skill [34]. Thus, there is a need to also emphasize other domains such as skills and emotion further so that the identified barriers are targeted for reduction in a future clinical trial.

Important aspects regarding the feasibility of this system were shown in this study. A promising result was the acceptability of the system to participants. Specifically, they noted no issue with comfort, a high motivation to wear it. However, they also noted poor usability according to the SUS. This is likely to be the result of their concern regarding the system and their ability to use it. Nonetheless, they demonstrated high levels of adherence and engagement with the tool compared with other research in HF or the population in general where abandonment is high [40-44], and importantly, they felt great comfort and reassurance that someone was looking out for them. Additionally, almost 50% of those contacted to take part in the study agreed to participate in it, suggesting that there was interest and acceptability in the idea. However, because of the purposive sampling method, it is unclear whether people with lower levels of literacy or higher levels of disease severity will demonstrate the same levels of acceptability. Among a sample of patients with HF, 96% owned a mobile phone and 32% relied on the mobile phone for internet access, searched health information, and reported moderate self-confidence in using mobile apps [3]; thus, the DHT should be tested in a wider population. However, in the future, when expanding this tool for use within multiple settings, the impact on HCPs needs to be considered. Specifically, the requirement for HCPs to monitor the triggers themselves, and follow up with patients, was perceived as an increased burden. In the future, HCPs whose role is to manage patient-driven data insights will be needed, but this will require additional funding and training to be implemented successfully. The volume of data required to train such models effectively was a surprise to the HCPs who had expected the system to be “smarter” than it was, demonstrating that there is a need to set expectations with HCPs in any future feasibility studies. This study was not powered or intended to assess the predictive capacity of the trigger system. Indeed, if comparing it with the Medical Research Council guidelines, this was the test phase required before evaluation [35]. However for future studies, models need to be refined until the thresholds per person are identified. Adjusting the threshold to become more manageable to HCPs should be considered, but within the context that participants valued feeling safe.

### Limitations

A key limitation of this study was that no objective assessment of participants’ behavior change (or lack thereof) was assessed. As a pilot study, the objective of this study was not to evaluate participants’ objective behavior change, and indeed, the study was not powered for that. Nonetheless, it limits our assessment of the potential for this DHT to patient perceptions only. Another limiting factor of this work is the recruitment methods employed, which are likely to have limited the generalizability of the findings with regard to representativeness of the sample. Specifically, purposive sampling was used whereby the HCPs contacted those on their list who they believed would be interested and capable of participating. Consequently, without objectively testing for health and digital literacy, the result of this is likely to be a bias toward people who were most likely to be able to use it. For DHTs to be evaluated fully, it must be offered to all, irrespective of their perceived suitability or not. Thus, future iterations of this tool should be trialed in as wide a range of participants as possible to test its ability to respond to patient needs and abilities. Finally, future studies need to assess feasibility more from the perspective of the health care service. Outcomes such as the number of trigger calls sent to the hospital, the time spent managing the data, the time spent by clinicians setting up the DHT with participants, and the number of visits to the clinic compared with the calls logged should be evaluated to better help understand the impact of the DHT on the service.

### Conclusions

The DHT demonstrated high levels of adherence and acceptance among participants. The DHT targeted key self-management behaviors and feelings of social support. However, a number of changes to the DHT, and the health service, are required before it can be implemented at scale. A full-scale feasibility trial conducted at a wider level is required to fully determine its potential effectiveness and wider implementation needs.

## Appendix

Multimedia Appendix 1 Interview protocol—participant.

Multimedia Appendix 2 Interview protocol—HCP. HCP: health care professional.

## Abbreviations

CRS: Comfort Rating Scale
DHT: digital health tool
EHFScBS: European Heart Failure Self-care Behavior Scale
HCP: health care professional
HF: heart failure
MLwHFQ: Minnesota Living with Heart Failure Questionnaire
SUS: System Usability Scale
TDF: Theoretical Domains Framework
WTMS: Wearable Technology Motivation Scale

## Data Availability

The data sets generated or analyzed during this study are not publicly available as we do not have ethical approval to share them. The anonymized questionnaires are available from the corresponding author on reasonable request.
