# Supplementary material for: Six-Month Pilot Testing of a Digital Health Tool to Support Effective Self-Care in People With Heart Failure: Mixed Methods Study
Source: JMIR Form Res. 2024 Mar 1;8:e52442. doi: 10.2196/52442 (PMC10959238; doi:10.2196/52442)
Supplement: Multimedia Appendix 1 [file formative_v8i1e52442_app1.docx]

# **Multimedia Appendix 1**

# Interview Protocol—Participant

This document outlines the topic guide/framework for the interviews with the research participants.

Methods

• All interviews will be recorded using a Dictaphone

• Subsequently, all interviews will be transcribed by the interviewer

• Once transcribed, the interviews will be coded using qualitative methods.

Interview Overview

Interviews will last approximately 1 hour and will take place at either the Beacon Hospital or University College Dublin. Interviews will follow the Topic Guide developed to capture data for interpretation and evaluation.

Aim

The aim of the interview is to explore the usability of the WISP heart app from the perspective of participants. Therefore, broadly, we wish to understand:

- How useful the app was to participants over the course of 6 months
- How easy it was to use
- How it influenced their daily lives
- How it influenced their emotions and thoughts regarding their condition
- Why they did/did not use it
- What needs to change to support them to use it more
- How did their behavior change as the months progressed

Interviews shall be semi-structured in nature. Specifically, there are a short number of included, open questions for all participants. If the participant is open and talks freely, they may answer some of the questions without being asked. Therefore, depending on the person, not all of these questions need to be asked.

If participants discuss anything regarding their condition, fears, and experiences of the app/overall WISP system, allow them to speak. Only interrupt them or alter the direction of the conversation when they stray from this broad area. if participants are not very open, some potential prompts have been included with the questions below. These prompts are there as an optional guide and do not need to be used.

General tips

When completing the interviews please remember the following:

- Start with broad questions to generate discussion.
- Try to avoid questions with multiple options as this will lead them towards a specific answer
- If you need to confirm specific details, then you can use questions which require short responses.
- Try to find a quiet area to complete the interview if possible. If possible, please also try to be alone with the participant. However, if they request or need to have another person present (e.g. carer, spouse) then do not refuse them this.
- Bring a notebook or something to take notes as you go. This will help you to remember key phrases. Participants may also tell you stories while they complete the questionnaires, you may write down these words at the time and ask about them in the interview.
- If participants remark that they are uncertain about something, please do not educate them during the interview. For example, if they remark that they do not know what the app was measuring, wait until the interview is complete before providing them with this information.

Topic Guide

**Can you describe your experience with the WISP Heart app during the past six months?**

**How did using the app impact your day to day life?**

**How did using the app make you feel about your condition?**

**What would need to change for you to use this app long-term?**

Note: For some participants we may need to explore what they typically do as part of understanding the usability of the app. If they start to go into their usual routine, or how they felt before the app, this is an acceptable direction to go in.
