# Supplementary material for: Six-Month Pilot Testing of a Digital Health Tool to Support Effective Self-Care in People With Heart Failure: Mixed Methods Study
Source: JMIR Form Res. 2024 Mar 1;8:e52442. doi: 10.2196/52442 (PMC10959238; doi:10.2196/52442)
Supplement: Multimedia Appendix 2 [file formative_v8i1e52442_app2.docx]

# **Multimedia Appendix 2**

# Interview Protocol—HCP

Interviews will last approximately 1 hour and will take place at either the Beacon Hospital or remotely via telephone. Interviews will follow the Topic Guide developed to capture data for interpretation and evaluation.

### Aim

The aim of the interview is to explore the usability of the WISP heart app. Therefore, broadly, we wish to understand:

- How useful the app was to participants
- How easy it was to use
- How it influenced their daily lives
- How it influenced their emotions and thoughts regarding their condition
- Why they did/did not use it
- What needs to change to support them to use it more

## Topic Guide

How did you find the experience of having the ECME system in place for the past six months?

Can you describe who was suitable for this system or not?

Can you describe how you usually support people in managing their heart failure?

How do you think this experience may differ from the public system?

At what stage of heart failure do you think this is most useful for and why?

How did the triggers impact your work?

What if anything might need to change to implement this in practice?
